# Afferent limb syndrome after total proctocolectomy and ileal pouch-anal canal anastomosis

**DOI:** 10.1186/s40792-020-00968-w

**Published:** 2020-08-14

**Authors:** Keiji Matsuda, Yojiro Hashiguchi, Kentaro Asako, Yuka Okada, Kohei Ohno, Mitsuo Tsukamoto, Yoshihisa Fukushima, Ryu Shimada, Tsuyoshi Ozawa, Tamuro Hayama, Keijiro Nozawa, Takeo Fukagawa, Yuko Sasajima

**Affiliations:** 1 Department of Surgery, Teikyo University School of Medicine, 2-11-1 Kaga, Itabashi-ku, Tokyo, Japan; 2 Department of Pathology, Teikyo University School of Medicine, Tokyo, Japan

**Keywords:** Ulcerative colitis, Afferent limb syndrome, Recurrent small bowel obstruction, Surgery, Ileopexy

## Abstract

**Background:**

Small bowel obstruction (SBO) is a common postoperative complication of ulcerative colitis (UC). There have been a few recent reports of afferent limb syndrome (ALS) as a rare occurrence in cases of SBO. We present a case of ALS with recurrent SBO that was successfully managed surgically.

**Case presentation:**

When this male patient was 55 years old, he underwent laparoscopy-assisted anus-preserving total proctocolectomy, the creation of a J-type ileal pouch, ileal pouch-anal canal anastomosis (IPAA), and creation of ileostomy for intractable UC. Three months later, ileostomy closure was performed. The first onset of SBO was observed 5 months after ileostomy closure. SBO occurred repeatedly, and the patient was hospitalized nine times in approximately 2 years. Each SBO was improved by non-surgical treatment. A computed tomography (CT) scan revealed that the afferent limb was narrowing and twisted, and gastrografin enema confirmed narrowing at the proximal portion of the pouch inlet. Endoscopy showed a sharp angulation at the pouch inlet. We suspected ALS and decided on a surgical policy and performed pouchopexy and ileopexy to the retroperitoneum by suturing with excision of the remaining blind end of the ileum. Endoscopy 3 days after surgery showed neither twist nor stricture in the fixed ileal pouch or the afferent limb. At the time of writing, the patient remains free of SBO symptoms.

**Conclusion:**

Clinicians should consider ALS when examining a patient with recurrent intermittent SBO after IPAA surgery. When ALS is suspected, the patient is indicated for surgery such as surgical pexy.

## Background

The surgical procedures for ulcerative colitis (UC) are total proctocolectomy and ileal pouch-anal (canal) anastomosis (IPAA). Small bowel obstruction (SBO) is a common postoperative complication of UC, with a reported frequency of 2–17.2% [1].

In 1997, as a peculiar cause of intestinal obstruction after IPAA, obstruction of passage due to flexion and torsion at the proximal portion of the ileal pouch was reported under the name of afferent limb obstruction [2]. Several similar cases followed and this type of obstruction came to be called afferent limb syndrome (ALS) [3]. Because of the characteristic finding that “no apparent stenosis is associated,” it is often difficult to make a diagnosis of ALS, and it is believed that there may be many hidden ALS cases in which SBO recurs without being diagnosed. It has been reported that the number of patients with UC is increasing [4], and it is clear that IPAA surgeries for UC are increasing [5]. The incidence of ALS is expected to increase in the future; however, it is very rare now. We present a case of ALS with recurrent SBO after IPAA, in which surgical management was effective.

## Case presentation

**Fig. 1:**
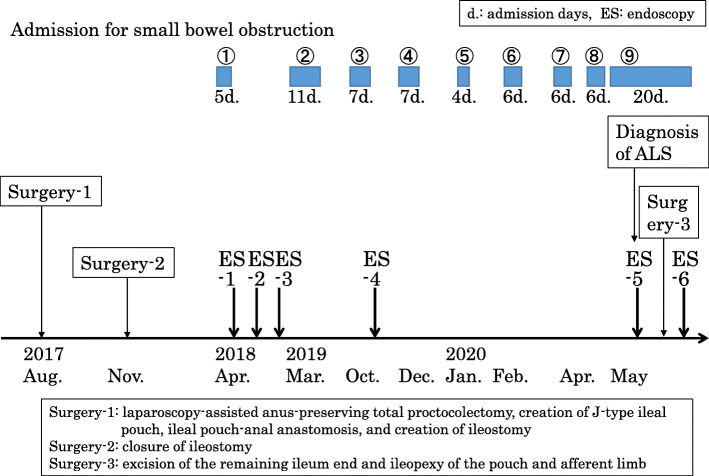
Clinical course after the patient’s first surgery for UC

A male patient developed UC at 33 years of age. As his UC was intractable to medical treatment including anti-tumor necrosis factor (TNF)α antibodies, he underwent laparoscopy-assisted anus-preserving total proctocolectomy, the creation of J-type ileal pouch, IPAA, and creation of ileostomy when he was 55 years old. Three months later, closure of ileostomy was performed with functional end-to-end anastomosis. The first onset of SBO was observed 5 months after ileostomy closure. SBO occurred repeatedly, and the patient was hospitalized nine times between April 2018 and May 2020 (Fig. 1). Each SBO was improved by non-surgical treatment and the patient’s hospital stays were relatively short, ranging from 4 to 11 days. Since he was hospitalized three times in 1 month (April to May, 2020), surgery was considered. He was a carrier of the hepatitis B virus and had a history of angina, from which he had recovered shortly before surgery. He had no family history of inflammatory bowel disease (IBD). He was 166 cm tall and weighed 52 kg, yielding a body mass index of 18.8 kg/m^2^. His laboratory data were as follows: hemoglobin, 13.2 g/dL (low); hematocrit, 37.3% (low); platelets, 25.8 × 10^4^/μl; white blood cells, 6800/μl (lymphocytes 14%, neutrophils 84%); albumin, 4.4 g/dL; and C-reactive protein (CRP), 0.07 mg/dL.

**Fig. 2:**
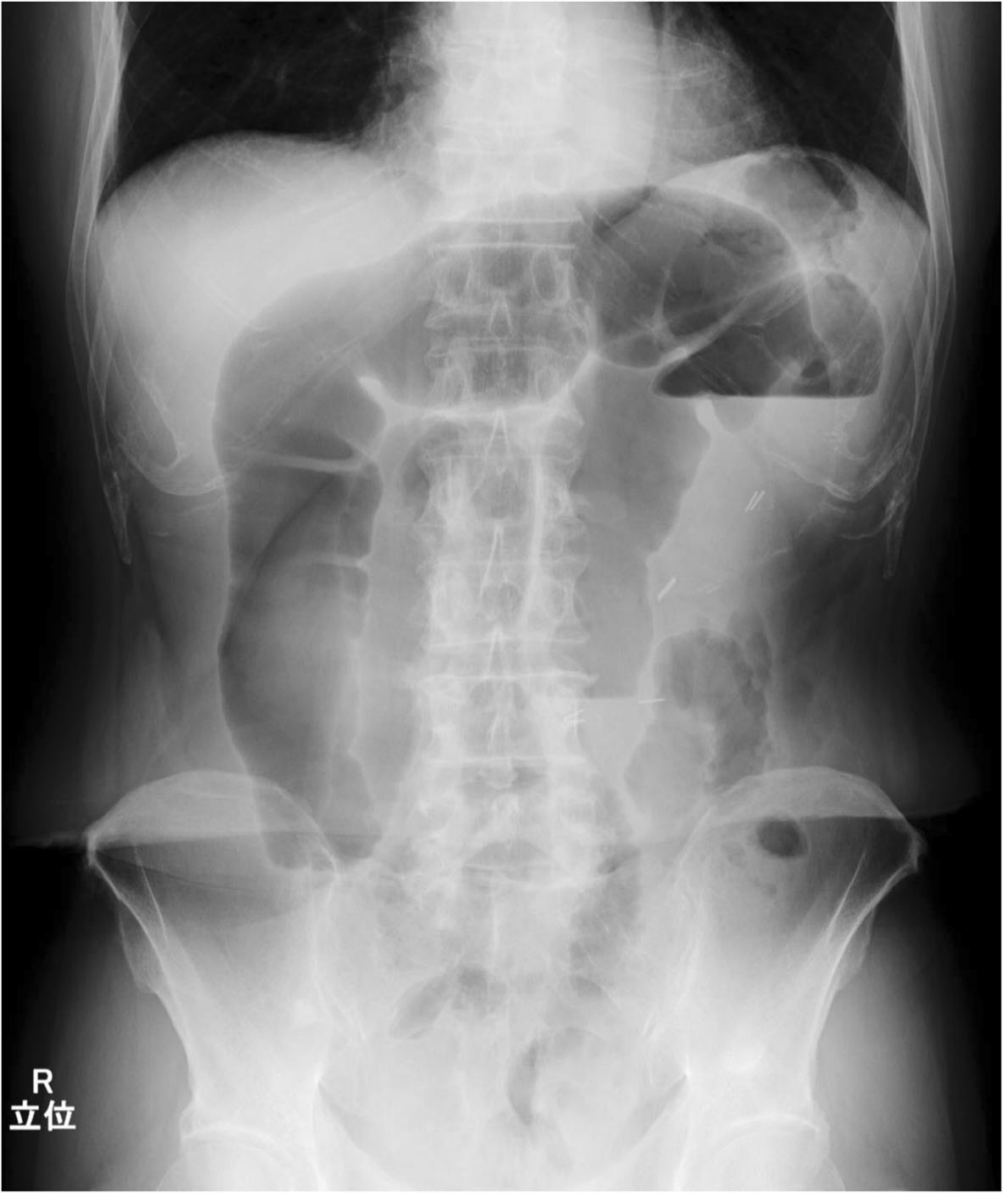
Intestinal gas was predominant on an abdominal X-ray

**Fig. 3:**
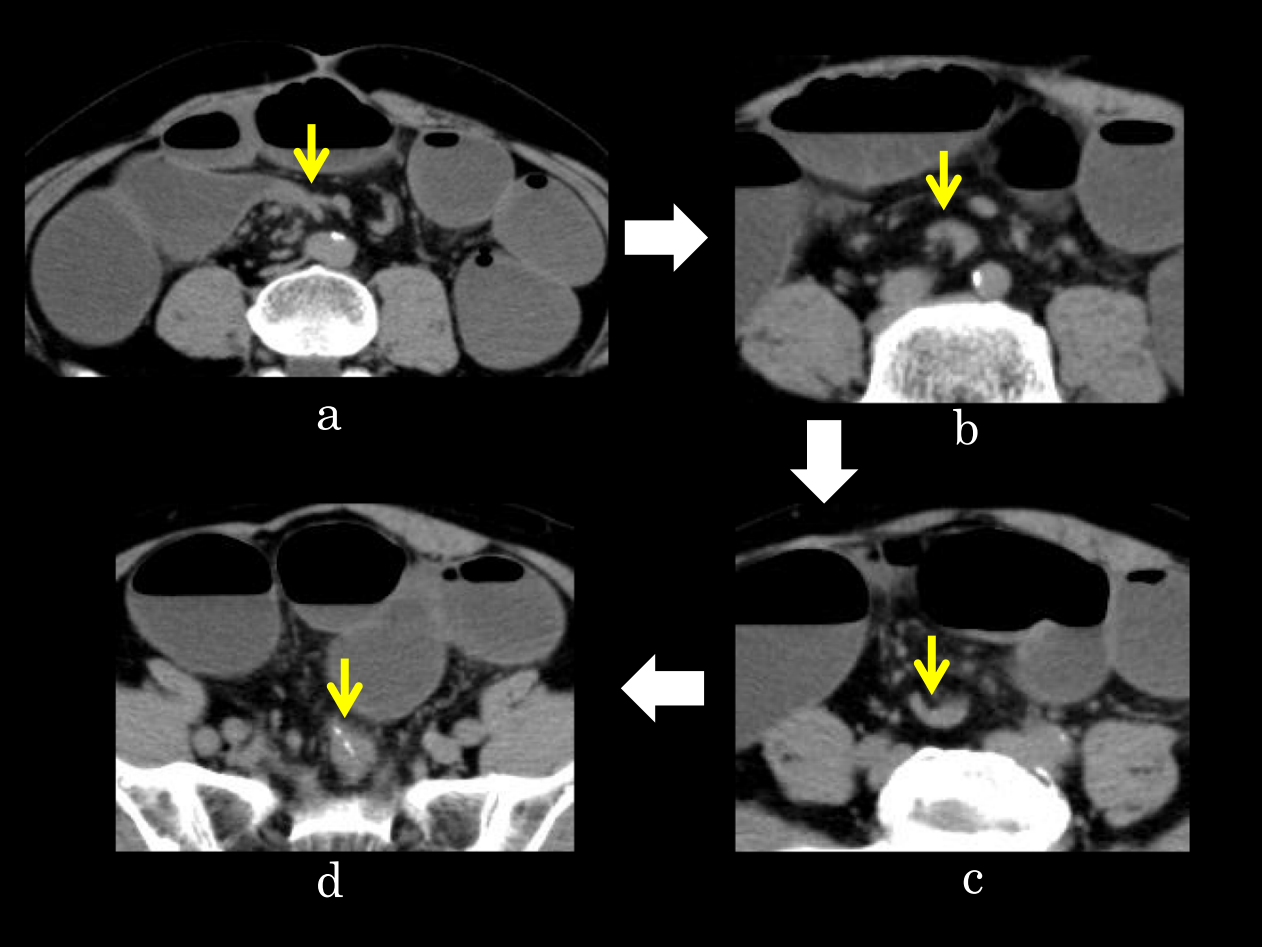
CT of the SBO. The ileal stricture had a beak-like appearance (**a**, yellow arrow). The ileum became narrower on the anal side and was twisted (**b**, **c**, yellow arrows), then it connected to the ileal pouch (**d**, yellow arrow)

**Fig. 4:**
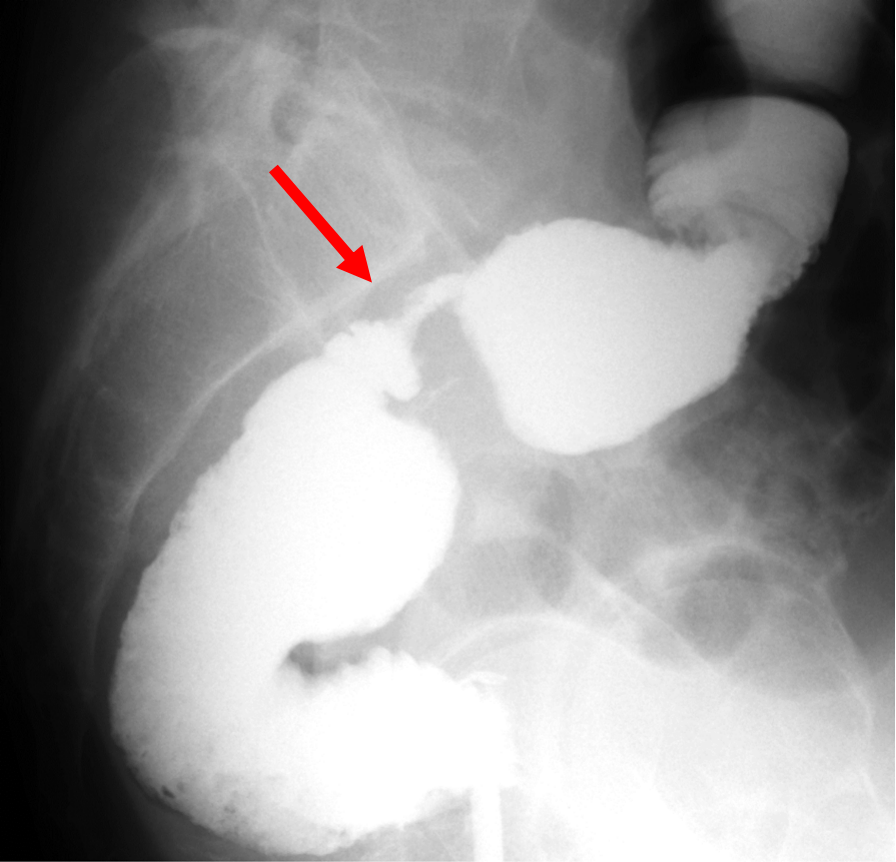
Gastrografin enema showed narrowing at the ileal pouch inlet (red arrow)

**Fig. 5:**
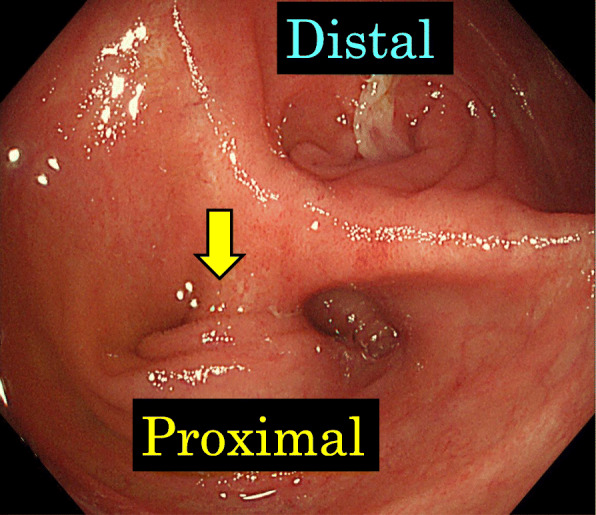
Endoscopy (ES-5) showed a sharp angulation at the inlet of the ileal pouch, and the proximal side could not be overlooked (yellow arrow)

**Fig. 6:**
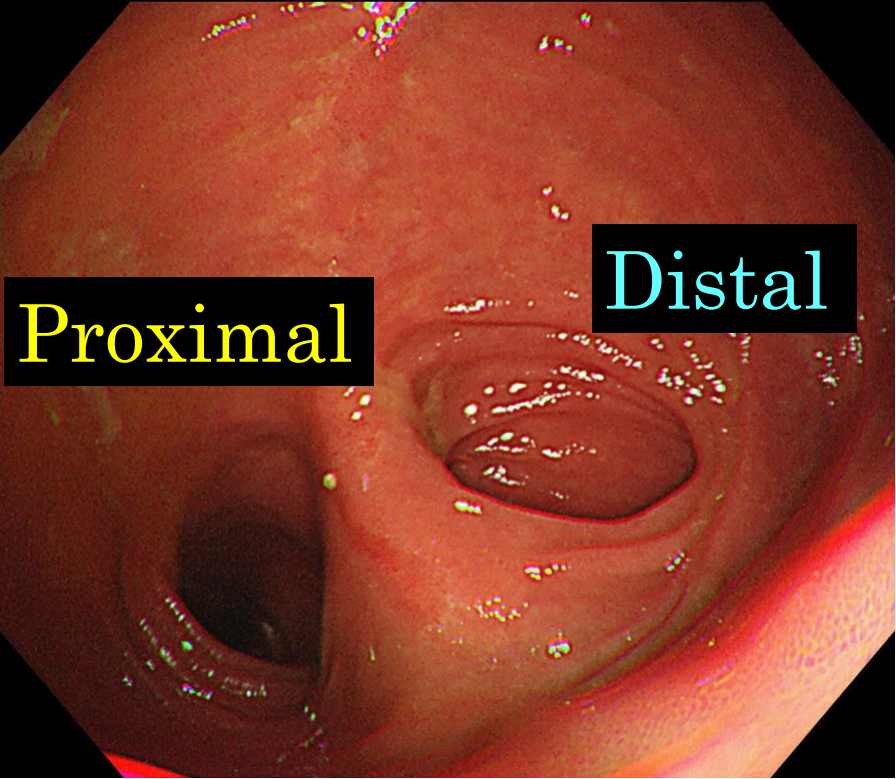
Endoscopy (ES-4) had shown that the pouch inlet was open without angulation

Intestinal gas was predominant on an abdominal X-ray (Fig. 2). A computed tomography (CT) scan revealed that the afferent limb on the proximal side of the ileal pouch was narrowing and twisted (Fig. 3). Gastrografin enema confirmed narrowing at the proximal portion of the pouch inlet (Fig. 4). Endoscopy in May of 2020 (Fig. 1, ES-5) showed a sharp angulation at the pouch inlet, but scope passage was not difficult (Fig. 5). After ileostomy, the patient underwent surveillance endoscopies for residual anal canal (Fig. 1, from ES-1 to ES-4). Surveillance endoscopy in November of 2019 (Fig. 1, ES-4) had shown that the pouch inlet was open without angulation (Fig. 6). Based on the above findings, we suspected ALS and decided on a surgical policy.

**Fig. 7:**
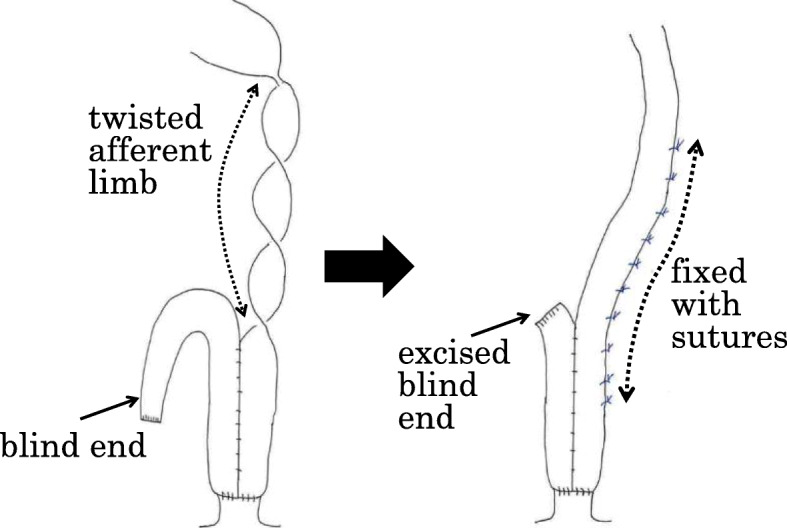
The blind end of the ileum was adhering to the pelvic floor. The remaining blind end of the ileum was excised. Since the ileal pouch and the afferent limb were easily twisted, we fixed them to the retroperitoneum with sutures

**Fig. 8:**
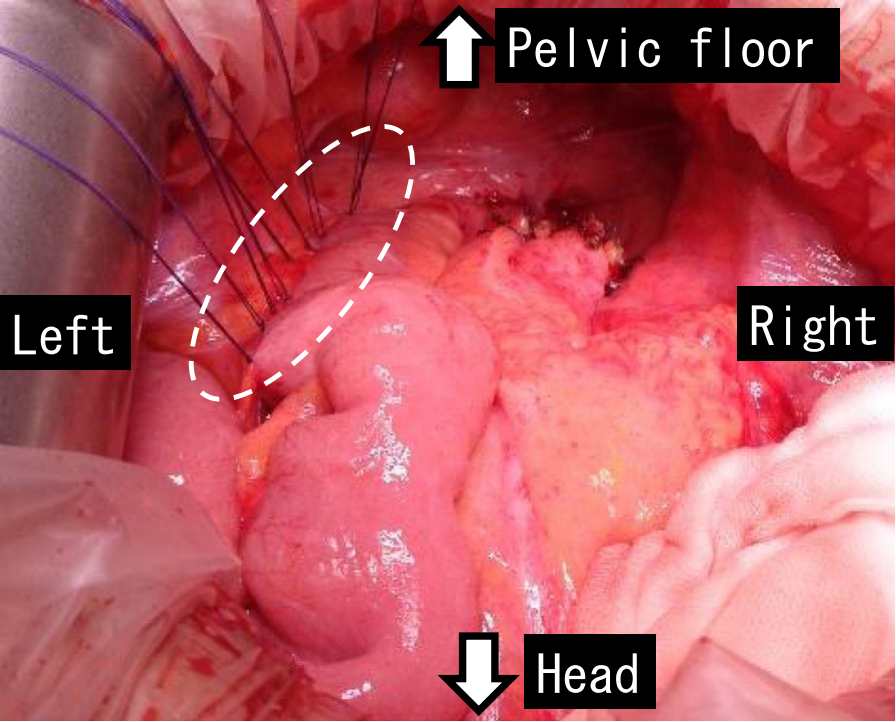
The ileal pouch and ileum of the afferent limb were fixed to the left retroperitoneum by suturing (white dotted circle)

Surgery was performed when the patient was 62 years old, 33 months after the IPAA surgery. The surgical method was as follows: a lower midline abdominal incision was made. No adhesion of the small intestine to the abdominal cavity was observed. The afferent limb was mobile, flexible, and easy to twist (Fig. 7). The blind end of the ileum, on the distal side from the pouch, was adhering to the pelvic floor. Because we could not rule out the cause of the intestinal obstruction because of the adhered blind end, we excised it. The remaining blind end of the ileum was excised by 15 cm. Since the ileal pouch and the afferent limb were easily twisted, we fixed them to the retroperitoneum with 10 sutures, using Coated 3-0 VICRYL® (ETHICON, NJ, USA), above the level of the iliac crest to prevent twisting (Fig. 8). A drain was inserted into the pelvis and the wound was closed.

**Fig. 9:**
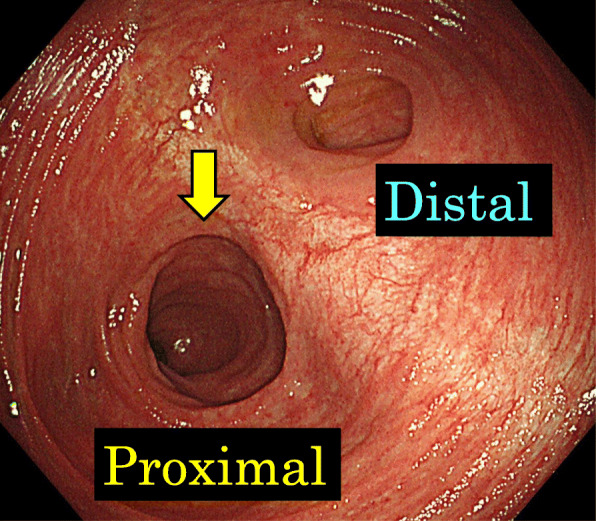
Endoscopy (ES-6) performed 3 days after surgery showed that the pouch inlet was wide open, and the afferent limb was clearly visible

The operative time was 2 h and 32 min, and blood loss was 48 ml. Postoperative paralytic ileus occurred, and endoscopy was performed 3 days after surgery (Fig. 1, ES-6). There was neither twist nor stricture in the fixed ileal pouch or afferent limb. The ileal pouch inlet was wide open, and the scope was easily inserted in the afferent limb (Fig. 9). Intestinal fluid and gas were aspirated as much as possible. The same procedure was performed the next day. Gas inhalation was performed twice by colonoscopy, and thereafter the treatment was unnecessary. The symptoms of ileus disappeared and the patients were discharged from the hospital 9 days after surgery. At the time of writing, 77 days passed since the surgery. The patient has suffered from neither abdominal pain nor fullness for 77 days postoperatively. And his weight has increased by 6 kg after surgery.

## Discussion

SBO after IPAA is a common postoperative complication, with a reported frequency of 2–17.2% [1]. ALS is a relatively new concept that was first reported in 1997 as occurring due to the loop of ileum just proximal to the pouch being trapped posteriorly between the pouch and sacrum [2]. Read et al. reported 6 cases, of which 2 underwent bypass surgery and 2 had pouchopexy [2]. Then, in a paper from the Cleveland Clinic, this condition was systematized and called ALS [6]. ALS was defined as sharp angulation leading to symptoms in any segment of the afferent limb from the pouch inlet to the previous loop ileostomy site, in the absence of intrinsic stricture [6]. This definition did not specify the loop being “trapped between the pouch and sacrum.”

The clinical symptoms of ALS have been reported, as have laboratory images and treatment methods. The clinical symptoms include dyschezia, straining, incomplete evacuation, and recurrent intermittent abdominal pain [3, 6, 7]. Our patient presented with recurrent intermittent abdominal pain and was hospitalized up to three times a month. Typical endoscopic findings are reported to include difficulties with intubation to the afferent limb during endoscopy due to the sharply angulated inlet. There should be no intrinsic stricture. In our patient, we could not see through the afferent limb because of the angulation of the entrance; however, insertion of the scope to the afferent limb during endoscopy was not difficult.

Radiographic findings are reported to include a minimum or a small quantity of contrast in the afferent limb. Management may be conservative, by adjusting the diet to small, frequent low-residue meals [6]. Endoscopy and contrast pouchogram are the main methods of diagnosis [3]. Surgical options include resection of the angulated bowel with anastomosis, lysis of the adhesion, surgical pexy of the pouch to the pelvic sidewall, pouch mobilization and small-bowel fixation, mesh placement, and pouch excision with end ileostomy [2, 3, 6]. Many researchers have recommended surgical pexy [2, 3, 6, 8–10]. In the present case, we performed surgical pexy of the pouch and ileum to the pelvic sidewall, which was effective for preventing SBO. No method has been reported to prevent ALS. In cases where afferent limb is thought to be prone to twisting after anus-preserving total proctocolectomy, suture fixation to the retroperitoneum might prevent afferent limb syndrome.

We searched Medline (using PubMed), Scopus, and the ISI Web of Knowledge for studies reporting cases of ALS associated with UC. The search terms included ulcerative colitis and afferent limb syndrome in all fields. Our search identified 9 documents. After examining the documents and their references, we summarized the reported cases of ALS in Table 1 [2, 8–11]. According to these data, the median age at the time of IPAA and at the time of the first operation for SBO was 40 years and 42.5 years, respectively. The median time between intestinal continuity and first SBO was 20 months, and 90% of patients needed to be admitted to the hospital more than once for SBO. Out of all 26 operations, 16 (62%) were ileopexy and/or pouchopexy, and the rate of postoperative recurrence of SBO was 19% (3/16).

**Table 1:**
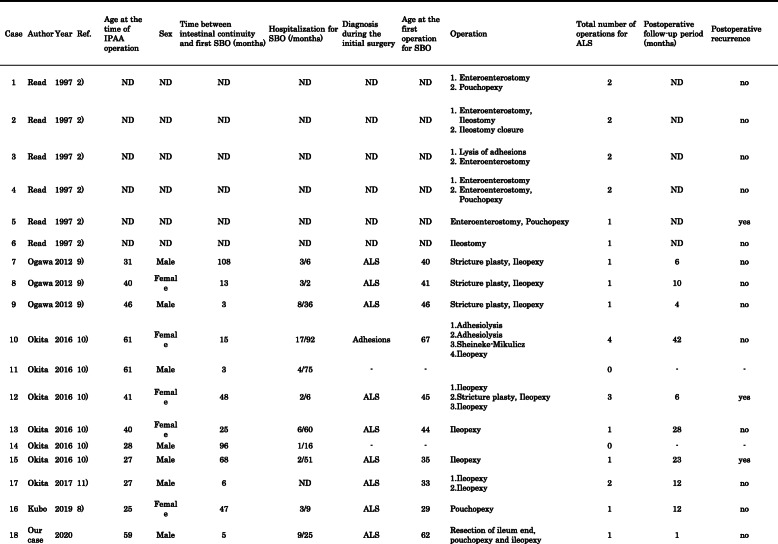
Reported case of afferent limb after IPPA operation

ALS afferent limb syndrome, IPAA ileal pouch anal (canal) anastomosis, ND no description, SBO small bowel obstruction

Recently, floppy pouch complex (FPC) has been proposed; this includes pouch prolapse, ALS, enterocele, redundant loop, and folding pouch [12]. Risk factors for FPC are reported to be a lowed body weight and a family history of IBD, the former of which would apply to our patient (52 kg; BMI, 18.8 kg/m^2^). Risk factors limited to ALS are expected to be derived in future study.

It is necessary to see the long-term progress of the present patient, but short-term results were extremely good. The patient had never suffered from abdominal symptoms and his weight has increased for 77 days postoperatively despite having being hospitalized 3 times in 2 months before the surgery.

## Conclusions

We have presented a case of successful surgical management of ALS, together with a summary of ALS cases reported to date. The diagnosis of ALS is difficult because there is no intrinsic stricture like adhesive ileus. Clinicians should consider ALS when examining a patient with recurrent intermittent SBO after IPAA surgery. When ALS is suspected, the patient is indicated for surgery such as surgical pexy.

## Data Availability

The authors declare that all of the data in this article are available within the article.

## Abbreviations

ALS: Afferent limb syndrome
CRP: C-reactive protein
CT: Computed tomography
FPC: Floppy pouch complex
IBD: Inflammatory bowel disease
IPAA: Ileal pouch-anal canal anastomosis
SBO: Small bowel obstruction
TNFα: Tumor necrosis factor alpha
UC: Ulcerative colitis
